# Supplementary material for: Opposing roles of nuclear receptor HNF4α isoforms in colitis and colitis-associated colon cancer
Source: eLife. 2016 May 11;5:e10903. doi: 10.7554/eLife.10903 (PMC4907689; doi:10.7554/eLife.10903)

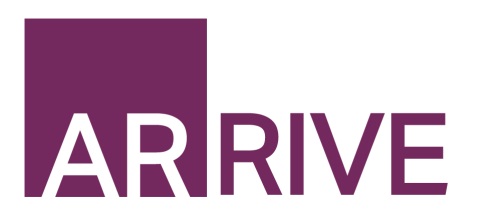


The ARRIVE Guidelines Checklist

Animal Research: Reporting In Vivo Experiments

Carol Kilkenny^1^, William J Browne^2^, Innes C Cuthill^3^, Michael Emerson^4^ and Douglas G Altman^5^

*^1^The National Centre for the Replacement, Refinement and Reduction of Animals in Research, London, UK, ^2^School of Veterinary Science, University of Bristol, Bristol, UK, ^3^School of Biological Sciences, University of Bristol, Bristol, UK, ^4^National Heart and Lung Institute, Imperial College London, UK, ^5^Centre for Statistics in Medicine, University of Oxford, Oxford, UK.*

|  | | ITEM | RECOMMENDATION | Section/ Paragraph |
| --- | --- | --- | --- | --- |
| 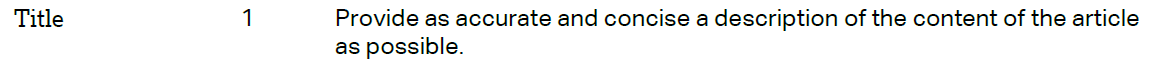 | | | Title |  |
| 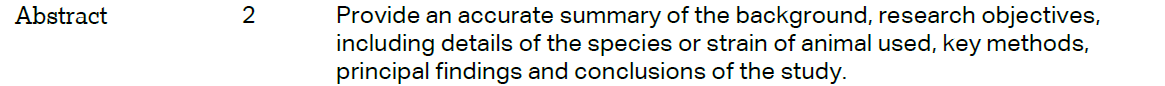 | | | Abstract |  |
| INTRODUCTION | | |  |  |
| 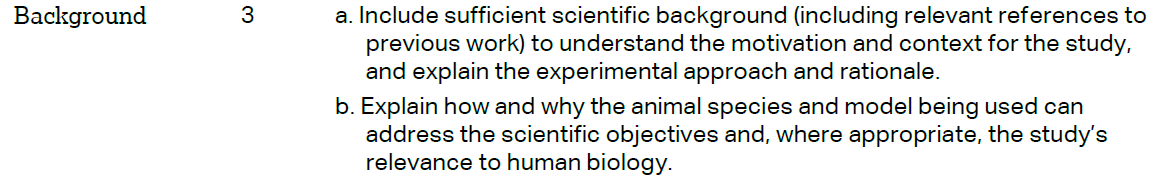 | | | Paragraphs 1-3 |  |
| 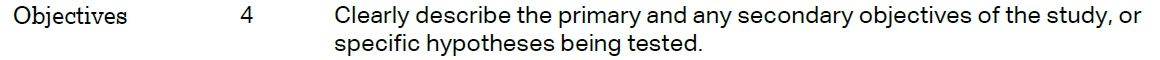 | | | Paragraph 4 |  |
| METHODS | | |  |  |
| 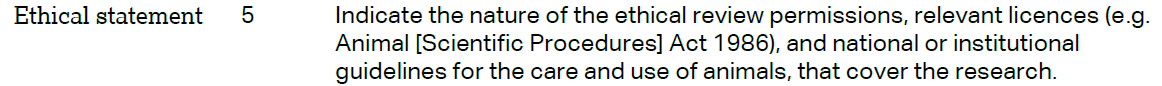 | | | Line 355-357; Institutional Protocol number A200140014 |  |
| 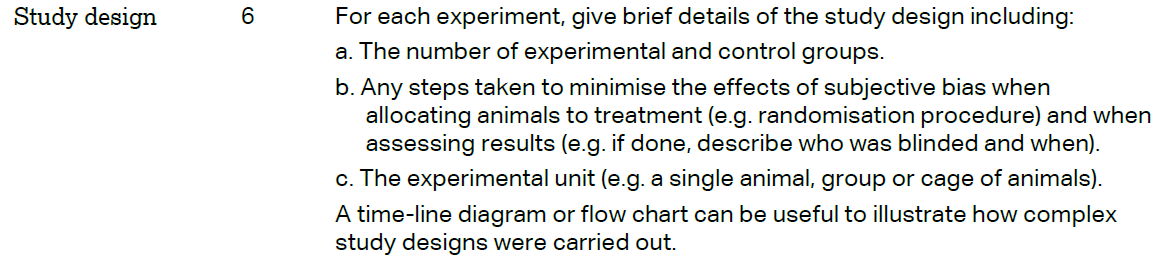 | | | a) Figure Legends  b) M&M |  |
| 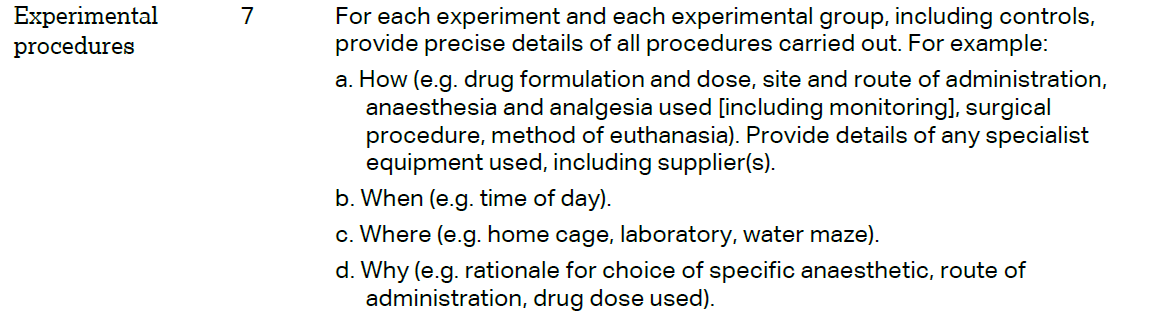 | | | Sections on Animal Use and Care, DSS colitis and Colitis-associated and sporadic cancer in M&M |  |
| 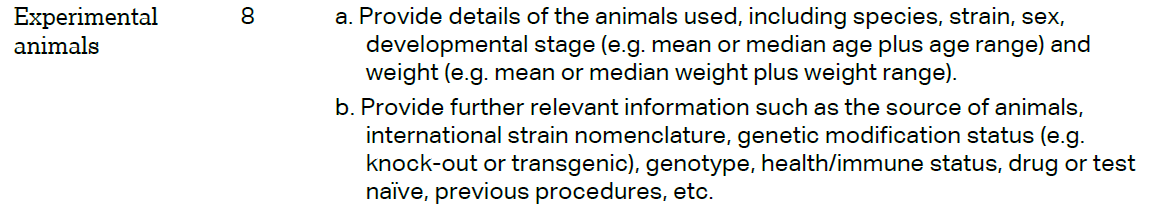 | | | Animal Use and Care section in M&M. |  |

The ARRIVE guidelines. Originally published in *PLoS Biology*, June 2010^1^

| 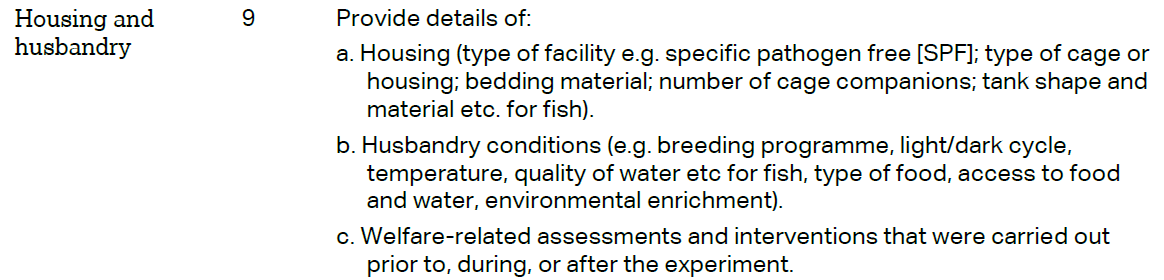 | Animal Use and Care and DSS colitis sections in M&M. | |
| --- | --- | --- |
| 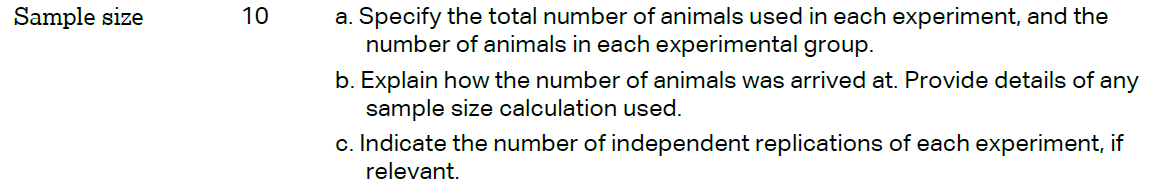 | a) Figure Legends  b) Statistical Analysis Assessment | |
| 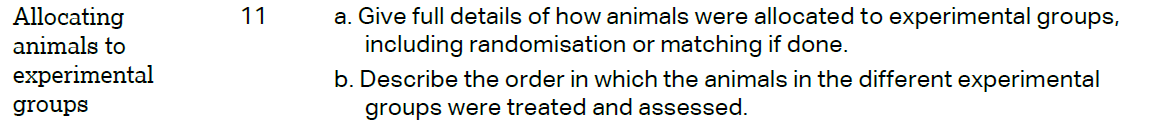 | a) Animal Use and Care in M&M  b) NA | |
| 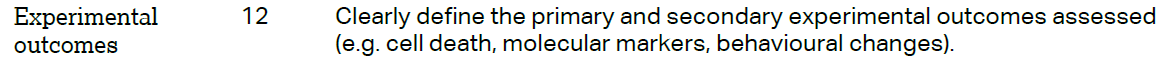 | Shown in figures | |
| 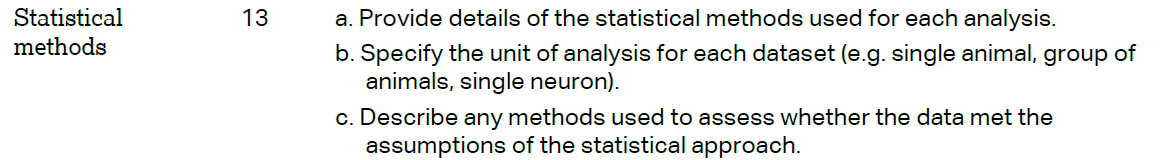 | Statistical analysis section in M&M | |
| RESULTS |  | |
| 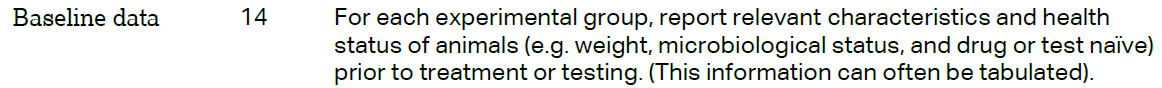 | Shown in figures for DSS. Tumor data: no baseline data needed – no tumors present | |
| 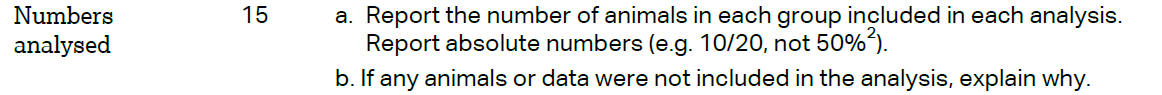 | Figure legends | |
| 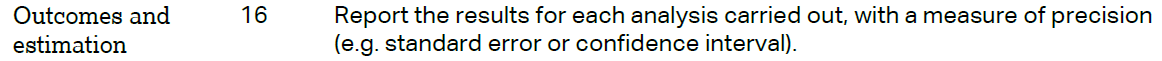 | Results section and Figures | |
| 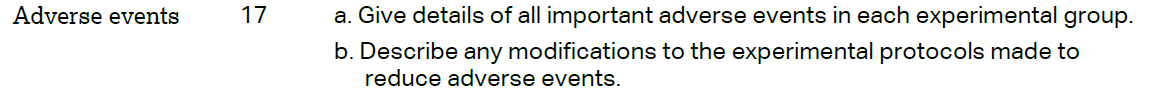 | See colitis and CAC section in M&M | |
| DISCUSSION |  | |
| 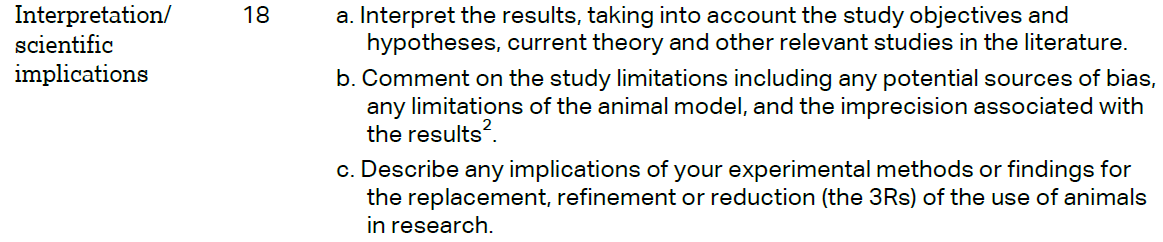 | Discussion | |
| 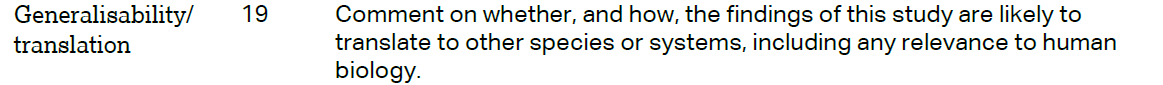 | Last paragraph of Discussion | |
| 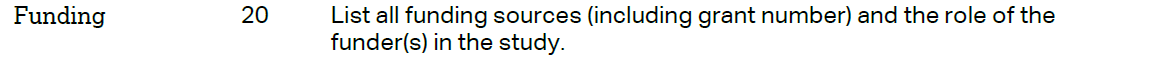 | | Info uploaded on website |


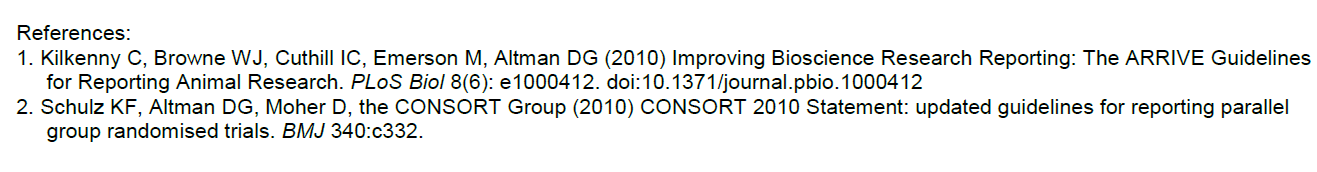

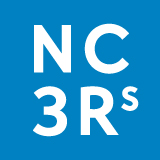

Supplement: Repoarting Standard. — DOI: http://dx.doi.org/10.7554/eLife.10903.018 [file elife-10903.docx]
